# Measurement properties of the brief resilient coping scale in patients with systemic lupus erythematosus using rasch analysis

**DOI:** 10.1186/s12955-016-0534-3

**Published:** 2016-09-13

**Authors:** José-Antonio López-Pina, Ana-Belén Meseguer-Henarejos, Juan-José Gascón-Cánovas, Dérlis-Julián Navarro-Villalba, Vaughn G. Sinclair, Kenneth A. Wallston

**Affiliations:** 1 Department of Basic Psychology and Methodology, Faculty of Psychology, Campus of Espinardo, University of Murcia, 30100 Murcia, Spain; 2 Department of Physical Therapy, University of Murcia, Murcia, Spain; 3 Department of Public Health, University of Murcia, Murcia, Spain; 4 Vanderbilt University, Nashville, USA

**Keywords:** Systemic lupus erythematosus, Resilient coping, Rasch analysis, Quality of life

## Abstract

**Bacground:**

Resilience has been defined as the capacity or the ability to rebound from and positively adapt to significant stressors, despite experiences of significant adversity or trauma. To capture to what extent an individual copes with stress in a resilient fashion the Brief Resilient Coping Scale (BRCS) was developed. This tool was validated in people with chronic disease, such as rheumatoid arthritis using standard psychometric techniques of classical test theory, but not yet in patients with Systemic lupus erythematosus (SLE). The aim of this study was to explore the psychometric properties of the Brief Resilient Coping Scale in patients with SLE using Rasch analysis.

**Method:**

This study used cross-sectional data. The BRCS was administered to 232 patients with systemic lupus erythematosus. The aspects analyzed were unidimensionality, local independence and differential item functioning (DIF) to construct an interpretative scale of scores with the Rasch model.

**Results:**

Rating scale mode (RSM) showed that the four categories used in the items of the BRCS are properly ordered. The four items provided a good fit to the polytomous Rasch model. Moreover, the parameters were sufficiently separated to measure resilience in patients with SLE. BRCS is a unidimensional scale (eigenvalue = 1.843) of resilience and the items were locally independent. There was no DIF between males and females in the sample. Only marginally significant differences depending on the level of education were found. The BRCS showed adequate discriminant validity between groups of scores.

**Conclusions:**

BRCS is a suitable scale for measuring resilience in patients with SLE. This scale might be useful for clinicians to obtain information concerning the degree of resilience that each patient has, allowing individuals with low resilience to be identified who need interventions aimed at developing coping skills.

## Background

Systemic lupus erythematosus (SLE) is a chronic autoimmune rheumatic disease, characterized by widespread inflammation of blood vessels and connective tissue [1]. The severity of symptoms, the secondary effects of medication, its unpredictability and early onset along with the chronic evolution of the disease are stress factors that provoke medium and long term psychological disorders in many sufferers [2, 3]. Such orders include anxiety and depression [4–7].

These may undermine the adaptation capacity of the patient, and their ability to maintain or regain mental health. This capacity of adaptation or facing up to the disease will largely depend on the patient’s psychological capacity, upon which is based the concept of resilience, which has been defined as the capacity or the ability to rebound from and positively adapt to significant stressors [8, 9], despite experiences of significant adversity or trauma [10].

Resilience was originally used in the field of physics to refer to the capacity of a material or systems to return to equilibrium after displacement, and has been adapted and developed in psychology as a theoretical construct of mental health protection, promotion and recovery processes [11]. Currently, this concept is increasingly used in the area of clinical medicine, especially, in patients with chronic diseases. Furthermore, some scientific research focuses on the role of resilience in chronic patients’ adaptation to their disease [12–14].

In this sense, in certain chronic diseases, such as cancer or Parkinson’s, patients with high levels of resilience have a better functional capacity, higher stability and better adaptation to their social environment. Also, the clinical symptomatology of these subjects is less severe, their pain threshold is higher, and they are less tired and are less likely to suffer from anxiety and depression, which leads to a better life quality [15, 16].

Because of this, evaluation of the degree of resilience is very important in subjects with chronic disease such as SLE, enabling deficit situations to be detected and improved [17, 18].

Resilience has been assessed mainly through self-report measures such as the Brief Resilient Coping Scale (BCRS), a 4-item measure that has been validated for people with rheumatoid arthritis, university students and ageing persons [19–21] using standard psychometric techniques of classical test theory. However, until now this scale has not been validated for people with SLE, nor has its psychometric properties been evaluated by item response theory [22].

For this reason, the aim of the present paper is to validate the Brief Resilient Coping Scale in patients with SLE using the Rasch model, testing unidimensionality, local independence, differential item functioning (DIF), and constructing an interpretative scale of scores obtained with the fitted model.

## Methods

### Participants and procedure

A cross-sectional study was developed in the province of Murcia (south-eastern Spain) with patients who met the revised American College of Rheumatology classification criteria for SLE diagnosis [23]. The BRCS and EQ-5D scales were administered, from July to August 2014, by postal survey to 450 eligible subjects selected randomly from the rare disease database of the Murcia Health Service. The study conforms to the principles of the Declaration of Helsinki [24] and was approved by the Research Bioethics Committee of the University of Murcia (Spain) (ID 1204/2015; 4/11/2015).

### Instruments

The Brief Resilient Coping Scale (BRCS) is a 4-item, unidimensional outcome measure designed to capture to what extent an individual copes with stress in a resilient fashion. In the original version, the items have a response format with five options, where 1 means the statement “does not describe you at all” and 5 means “it describes you very well”, but in this work the number of categories was reduced to four because the central category was removed to force decision-making of patients (Appendix). The BRCS meets the minimal standard for reliability and validity of a resilience instrument. In this study, the internal consistency coefficient was 0.82 and the Spearman-Brown coefficient was 0.81. The concurrent validity coefficient for BRCS was 0.34 with EQ-5D and 0.34 with EQ-VAS.

The EuroQol (EQ-5D) is a generic health index which comprises a five-part questionnaire (mobility, self-care, usual activities, pain/discomfort, anxiety/depression) to calculate a ‘utility’ or health index value between “0” and “1”, and a visual analogue self-rating scale (EQ-VAS) which ranges from 0 (minimum score) to 100 (maximum score). The EQ-5D is a reliable and valid instrument for measure quality of life [25, 26].

### Rasch analysis

The Rasch family of models transforms ordinal scores to interval scales (logits) [27]. For this it is necessary to specify a model according to the structure of the items in the scale. If the data fit the model specified, then independent item and ability parameters will be estimated. Also, applying the Rasch model involves evaluating the order of the item categories, fitting of the items, the separability of the parameters, and DIF. If all the analyses indicate that the scale forms a unidimensional rule of resilience, it will be possible to construct a more interpretative transformed scale.

There are two models in the Rasch family of models to estimate the location parameters of the items: the partial credit model (PCM) [28] and the rating scale model (RSM) [29]. Both models determine the parameters of the transitions between the categories of the items, but PCM allows each item to have a different unordered threshold, while RSM permits equal transitions between categories for each item. Therefore, a first evaluation of the appropriate model to explain the BRCS results involves examining whether the average score of the people who have answered each category in each item increases monotonically. A second evaluation involves comparing the fit in both models with the deviance (G^2^) which follows an approximately normal distribution with *df* equal to the difference between the free parameters estimated in each of the models. If the difference between deviances in both models is not significant at *p* < .05, then the simplest model is selected to explain the matrix of responses to the BRCS.

To estimate the parameters of the Rasch models ConQuest v. 3.0 [30] was used, and for descriptive statistics and convergent validity we used SPSS v. 19.0.

### Item fit

To assess the goodness of fit of the data to the Rasch model Conquest uses two statistics based on residuals of mean square (MNSQ). The unweighted mean square detect unexpected responses pattern when ability parameter is far to the item location parameter, while weighted mean square detect unexpected responses when ability parameter is very close to the item location parameter. An item shows a good fit to Rasch models if the unweighted or weighted item fit is found in the interval 0.6 to 1.4 [31].

### Reliability

Conquest also provides a separability coefficient of the parameters that makes it possible to evaluate whether the localization parameters of the items are sufficiently separate to cover the whole interval of the ability. In this case, the parameters are separate enough if the reliability coefficient is equal to or higher than 0.90 or *χ*^2^ is significant.

### Unidimensionality and local independence

In the Rasch model the items must form a unidimensional scale. Unidimensionality was tested with a principal components analysis (PCA) of residuals. It was considered that the BRCS was unidimensional if at least the first principal component explained a 50 % of the variance and the eigenvalue was higher than 1.5. Local independence was examined with inter-item residual correlation matrix. The items were locally independent if inter-item residual correlations was lower than 0.70.

### DIF

DIF is evidence that the probabilities of the response of the groups can vary through the continuum of ability. Therefore, DIF exists if a group has a higher probability of offering an answer rather than another one systematically though all the levels of ability. In the present study DIF has been tested with regard to gender, a difference between male and female no greater than 0.50 has been taken as a criterion of non-DIF [32].

### Wright map

A Wright map [27] allows ability parameters to be compared with item localization parameters on the BRCS in terms of logits. The Wright map to assess whether the calibration sample is appropriate for the group of items selected and has been useful or not. In both cases, this imbalance would cast doubt on the results of the calibration of the scale.

### Differences between groups

After fitting the data to the Rasch model, the differences in resilience as regards gender, education and age interval were determined by Student’s *t* test or ANOVA, depending on the number of levels of each independent variable. Statistical significance was taken as *p* < 0.05.

### Convergent validity

To explore convergent validity we hypothesized that resilience would be higher among those SLE patients who have a greater quality of life, as shown by literature studies [33–35]. Subjects were first divided into 3 categories (low, moderate, high) using tertiles of HRQoL level as cut-off points. We then used ANOVA followed by the MSD *post hoc* test to compare mean scores from the BRCS across these 3 categories as a way to infer discriminant ability regarding HRQoL.

## Results

Age, gender, disease duration, educational level and comorbidity with other illness in the sample appear in Table 1. The sample consisted of 232 cases (88.2 % females) who returned their postal survey, a response rate of 51.5 %. The average age was 48.6 years (SD = 13.3) and the average duration of the illness was 15.9 years (SD = 9.1).

**Table 1:** Patients’ characteristics (*N* = 232)

	Mean (SD)	N(%)
*Age* (years)	48.6(13.3)	
*Disease duration* (years)	15.9(9.1)	
*Gender*		
Female		202(88.2)
Male		30(11.8)
*Education*		
No schooling		22(9.7)
Primary Education		86(37.9)
Secondary Education		75(33.0)
Higher Education		44(19.4)
*Comorbidity*		
Osteoporosis		58(25.3)
Depression		88(38.4)
Diabetes		23(10.0)
Osteoarthritis		63(27.5)
Anaemia		87(38.0)
Peripheral vascular desease		60(26.2)
Vertebral fractures		20(8.7)
Kidney failure		60(26.2)
Lung failure		45(19.7)

### Ordering of categories

Table 2 shows the mean and typical deviation of the item scores regarding the categories. The gradual increase of the means in each category signifies that the categories are well ordered and no unexpected violations have occurred.

**Table 2:** Frequencies(%) distribution for each item of the Brief Resilient Coping Scale

	Categories			
Item	0	1	2	3
I look for creative ways to alter difficult situations.	19(8.3 %)	55(24.1 %)	78(34.2 %)	76(33.3 %)
*Average score within category*	1.47	4.53	7.18	10.34
*SD Score within category*	1.58	1.64	1.62	1.54
Regardless of what happens to me, I believe I can control my reaction to it.	39(17.1 %)	69(30.3 %)	78(34.2 %)	42(18.4 %)
*Average score within of category*	2.87	5.51	8.71	10.76
*SD Score within of category*	2.03	1.86	1.64	1.56
I believe i can grow in positive ways by dealing with difficult situations	28(12.3 %)	65(28.5 %)	82(35.9 %)	53(23.3 %)
*Average score within of category*	2.00	5.38	7.83	10.85
*SD Score within of category*	1.66	1.83	1.70	1.42
I actively look for ways to replace the losses I encounter in life.	16(7.0 %)	49(21.5 %)	93(40.8 %)	70(30.7 %)
*Average score within of category*	1.75	4.35	7.01	10.43
*SD Score within of category*	2.24	1.83	1.87	1.57

### Which model is better?

Which is the best model to explain the response matrix was tested by comparing deviance of PCM vs. RSM. Deviance for PCM was 1954.767 with *df* = 13, and for RSM was 1957.619 with *df* = 7. The difference was 2.852 (*df* = 6), which was not statistically significant (*p* > 0.05). So, RSM was chosen as the most appropriate model for the BRCS. The location parameters appear in Table 3.

**Table 3:** Parameters of location and fitting statistics for the items and thresholds parameters of the Brief Resilient Coping Scale

				Unweighted fit			Weighted fit		
Item	Location parameter	Standard error	R_jX_	MNSQ	CI	T	MNSQ	CI	T
1	−0.431	0.082	.87	0.87	[0.82, 1.18]	−1.4	0.95	[0.81–1.19]	−0.6
2	0.706	0.081	.83	1.11	[0.82, 1.18]	1.2	1.09	[0.82, 1.18]	0.9
3	0.237	0.081	.85	0.94	[0.82, 1.18]	−0.6	0.96	[0.82, 1.18]	−0.5
4	−0.512^a^	0.141	.83	0.98	[0.82, 1.18]	−0.1	1.01	[0.81, 1.19]	0.1
Categories									
0				0.94	[0.82, 1.18]	−0.7	1.11	[0.70, 1.30]	0.7
1	−2.30	0.083		1.24	[0.82, 1.18]	2.4	1.23	[0.80, 1.20]	2.1
2	−0.81	0.077		1.25	[0.82, 1.18]	2.5	1.27	[0.82, 1.18]	2.7
3	2.39^a^			1.12	[0.82, 1.18]	1.3	1.16	[0.77, 1.23]	1.3

*Notes*: ^a^the parameter is constrained, *R*_*jX*_ item-test corrected correlation, *MNSQ* Mean square, *CI* Confidence Interval, *T* Transformation Wilson-Hilferty

**Fig. 1:**
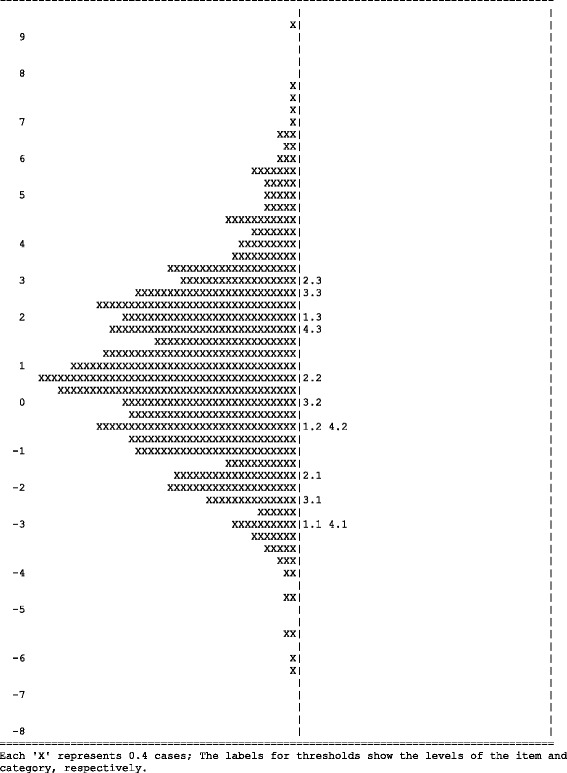
Wright map for BRCS

As expected from a scale constructed based on the classic test model, the four items had high homogeneity (0.83 to 0.87) and the localization parameters were very close to the mean resilience of the sample used during the study. Items 2 and 3 were indicative of high resilience, while items 1 and 4 were below average parameters. The thresholds between categories were in order and sufficiently separated, indicating that they fulfilled their expected function of representing the degree of resilience in this sample, as shown in the Wright map (Fig. 1).

### Item fit

Both the unweighted and weighted mean square for the items and the categories were in the specified interval [0.6, 1.4], so it is safe to say that the responses on the BRCS scale follow the RSM (Table 3).

### Separation reliability coefficient

The separation coefficient of the parameters was .98 (*χ*^2^ = 112.3, *df* = 3, *p* < 0.001), which indicates that the thresholds of the four items were able to cover the interval of resilience of the sample in which the scale was calibrated, in spite of the small number of items of the BRCS.

### Dimensionality and local independence

Dimensionality of the BRCS was tested with a residual PCA. The small number of items of the scale prevented the first factor from obtaining the expected salience as in other larger scales, but the first main component explained 46.08 % of total variance (eigenvalue = 1.843) so the scale was considered to be essentially unidimensional.

Local independence was obtained through correlations of the standardised residuals of the items which varied between −0.06 and −0.49, never reaching the limit of 0.70, so that the responses to the items were also considered to be locally independent.

### DIF

The difference between males and females in the localization parameters of the four items was 0.11, never surpassing 0.50, so DIF was not significant for any item.

### Differences between groups

After fitting the BRCS to RSM, the resilience parameters for the sample were estimated. No significant differences were found with respect to gender or age interval, but marginally significant differences (*p* = .061) were found between educational levels, the mean of the resilience parameters increasing with educational level. Thus, the group of patients who had only studied primary education obtained a mean of resilience of 0.146 (SD = 2.69), while those who had received higher education obtained a mean of 1.307 (SD = 2.40) (Table 4).

**Table 4:** Differences between groups (Student’s t /ANOVA)

	Mean (SD)	*t/F*	*df*	*P*
Gender				
Male	1.28 (2.07)	1.10	226	0.271
Female	0.74 (2.39)			
Educational level				
No schooling	0.15 (2.69)	2.50	(3, 226)	0.061^a^
Primary education	0.40 (2.40)			
Secundary education	1.01 (2.08)			
Higher education	1.31 (2.40)			
Age Interval				
1 (<45 años)	0.93 (2.36)	.22	(2, 226)	0.800
2 (45–65 años)	0.75 (2.22)			
3 (>65 años)	0.63 (2.70)			

^a^Marginally significant

### Convergent validity

Table 5 presents the BRCS scores by HRQoL level. Scores increased significantly with increasing either EQ-5D or EQ-VAS levels. The mean score obtained by the group of patients with a high level of EQ-VAS (60.2 to 100) was 69.7, which is 39.4 % per cent higher than the low-level category (*p* < 0.001), these differences were also observed when comparison between extreme groups was made using the “utility” values obtained by means of EQ-5D (41.2 %) providing, in both cases, evidence of the construct validity of the BRCS.

**Table 5:** Convergent validity of the Brief Resilient Scale (BRCS) regarding HRQol (Euroqol test)^a^

	BRCS Scores	
	n	Mean [0–100] (CI 95 %)
*EQ visual analogue scale (EQ-VAS)Tertiles*		
Low (T1) [≤40.0]	75	50.9 (45.2-56.6)
Moderate (T2) [40.1–60.1]	66	60.1 (54.1-66.1)
High (T3) [60.2–100.0]	69	69.7 (62.9-76.5)
Overall mean	210	60.0 (56.3-63.7)
*EQ-5D descriptive system Tertiles*		
Low (T1) [≤0.08]	75	49.1 (43.5-54.7)
Moderate (T2) [0.08–0.31]	77	60.2 (54.2-66.2)
High (T3) [0.32–1.00]	73	69.5 (63.3-75.7)
Overall mean	225	59.5 (55.9-63.1)

^a^One-way analysis of variance followed by post hoc test. *EQ* EuroQol, EQ-VAS: Ho: T1 *vs* T2= > *p* = 0.03; Ho: T1 *vs* T3= > <0.001: Ho: T2 *vs* T3= > *p* = 0.03. EQ-5D: Ho: T1 *vs* T2= > *p* = 0.009; Ho: T1 *vs* T3= > <0.001: Ho: T2 *vs* T3= > *p* = 0.02

## Discussion

This is the first study that gives evidence of the reliability and validity of the BCRS using Rasch analysis, in a sample of patients with SLE. Good support was established for the psychometric properties of the BRCS with a good fit to the RSM, and no DIF was found. There was good internal consistency and support for the unidimensionality and local independence of the items on the scale. These results are in agreement with other produced with classical test theory [19–21].

The studies made to date in which resilient coping has been studied in patients with SLE have used the resilience scale (RS) [36, 37]. This scale has 25 items and was developed for the general population. However, the brevity of the BCRS allows it to be completed quickly and easily by patients with conditions such as SLE, and that it can be administered multiple times in longitudinal studies as well as in large surveys. Validation of this scale using the Rasch model also points to its potential usefulness in daily clinical practice in patients with systematic erythematosus lupus.

Study of the dimensionality showed that the BRCS forms a unidimensional scale with localization parameters of the items and thresholds of the categories which clearly separate the resilience of the evaluated patients. There was no evidence of local dependence in the answers to the items.

This scale reflects one of the patterns of resilience, more specifically the situational pattern, which corresponds to resilient coping patterns [10]. Items in this measure refer to tenacity, optimism, creativity, and aggressive approach to problem solving, and commitment to positive growth from difficult situations [19]. By administering this scale to the patients, we obtain information concerning the degree of resilience that each patient has, allowing individuals with low resilience to be identified who need interventions aimed at developing coping skills. In this way, patients who receive treatment will develop abilities to face stressing situations daily, lessening the possibility of a psychological crisis. In fact, in the study of Sinclair & Wallston [19] where BRCS was validated in a sample of rheumatoid arthritis in the US, it was observed that resilient coping showed significant improvement after a cognitive-behavioral intervention designed to enhance adaptive coping, which indicates that levels of resilient coping can be improved.

In addition the BRCS correlates with perceived health status in patients with SLE, providing evidence of its construct validity. If interventions to build resilient coping could be refined, then perhaps quality of life could be improved in this population with a stressful chronic condition. The improvement of the quality of life in an incurable chronic illness is a primary objective and very important for patients. It would be advisable to consider cognitive-behavioral interventions aimed at enhancing resilient coping as a no-pharmacological treatment important for patients with SLE. This recommendation has been suggested in a study with Parkinson’s patients, where resilient coping is considered one of the determining factors of disability and quality of life [16].

The length of the scale, only 4 items, may be considered a limiting factor of the quality of the scores, although its brevity means it can be used in clinical contexts where patients might not answer other longer resilience scales (“short but sweet”).

Future research will study the properties of the BRCS in other clinical samples with chronic (e.g., fibromyalgia, diabetes, atopic dermatitis) or degenerative disease (e.g., Parkinson’s disease, Multiple sclerosis).

## Conclusions

The BRCS is a suitable scale for measuring resilience in patients with SLE. This scale might be useful for clinicians can obtain information concerning the degree of resilience that each patient has, allowing individuals with low resilience to be identified who need interventions aimed at developing coping skills.

## Appendix

**Table 6:** BRC scale

*Consider how well the following statements describe your behavior and actions*				
1. I look for creative ways to alter difficult situations.	0	1	2	3
	Does not describe me at all	Does not describe me	Describes me	Describes me very well
2. Regardless of what happens to me, I believe I can control my reaction to it.	0	1	2	4
	Does not describe me at all	Does not describe me	Describes me	Describes me very well
3. I believe that I can grow in positive ways by dealing with difficult situations.	0	1	2	3
	Does not describe me at all	Does not describe me	Describes me	Describes me very well
4. I actively look for ways to replace the losses I encounter in life.	0	1	2	3
	Does not describe me at all	Does not describe me	Describes me	Describes me very well

## References

[1] Iverson GL (1995). The Need for Psychological Services for Persons with Systemic Lupus Erythematosus. Rehabil Psychol.

[2] Hale ED, Treharne GJ, Lyons AC, Norton Y, Mole S, Mitton DL (2006). “Joining the dots” for patients with systemic lupus erythematosus: personal perspectives of health care from a qualitative study. Ann Rheum Dis.

[3] Burckhardt CS, Archenholtz B, Bjelle A (1993). Quality of life of women with systemic lupus erythematosus: a comparison with women with rheumatoid arthritis. J Rheumatol.

[4] Jennekens FG, Kater L (2002). The central nervous system in systemic lupus erythematosus. 1. Clinical syndromes: a literature investigation. Rheumatology (Oxford).

[5] Seawell AH, Danoff-Burg S (2004). Psychosocial research on systemic lupus erythematosus: a literature review. Lupus.

[6] Segui J, Ramos-Casals M, García-Carrasco M, de Flores T, Cervera R, Valdés M (2000). Psychiatric and psychosocial disorders in patients with systemic lupus erythematosus: a longitudinal study of active and inactive stages of the disease. Lupus.

[7] Petri M, Naqibuddin M, Carson KA, Wallace DJ, Weisman MH, Holliday SL (2010). Depression and cognitive impairment in newly diagnosed systemic lupus erythematosus. J Rheumatol.

[8] Dyer JG, McGuinness TM (1996). Resilience: Analysis of the concept. Arch Psychiatr Nurs.

[9] Bonanno GA, Wortman CB, Lehman DR, Tweed RG, Haring M, Sonnega J (2002). Resilience to loss and chronic grief: a prospective study from preloss to 18-months postloss. J Pers Soc Psychol.

[10] Masten AS (2001). Ordinary magic-resilience processes in development. Am Psychol.

[11] Davydov DM, Stewart R, Ritchie K, Chaudieu I (2010). Resilience and mental health. Clin Psychol Rev.

[12] Braun A, Müller UA, Müller R, Leppert K, Schiel R (2004). Structured treatment and teaching of patients with type 2 diabetes mellitus and impaired cognitive function-the DICOF trial. Diabetes Med.

[13] Hou WK, Law CC, Yin J, Fu YT (2010). Resource loss, resource gain, and psychological resilience and dysfunction following cancer diagnosis: a growth mixture modeling approach. Health Psychol.

[14] Aspinwall LG, MacNamara A (2005). Taking positive changes seriously: toward a positive psychology of cancer survivorship and resilience. Cancer.

[15] Schumacher A, Sauerland C, Silling G, Berdel WE, Stelljes M (2014). Resilience in patients after allogeneic stem cell transplantation. Support Care Cancer.

[16] Robottom BJ, Gruber-Baldini AL, Anderson KE, Reich SG, Fishman PS, Weiner WJ (2012). What determines resilience in patients with Parkinson’s disease?. Parkinsonism Relat Disord.

[17] Luthar SS, Cicchetti D (2000). The construct of resilience: Implications for interventions and social policies. Dev Psychopathol.

[18] Polk LV (1997). Toward a middle-range theory of resilience. Adv Nurs Sci.

[19] Sinclair VG, Wallston KA (2004). The development and psychometric evaluation of the Brief Resilient Coping Scale. Assess.

[20] Tomás JM, Meléndez JC, Sancho P, Mayordomo T (2012). Adaptation and initial validation of the BRCS in an elderly Spanish sample. Eur J Psychol Assess.

[21] Limonero JT, Tomás-Sábado J, Gómez-Romero MJ, Maté-Méndez J, Sinclair VG, Wallston KA (2014). Evidence for validity of the brief resilient coping scale in a young Spanish sample. Span J Psychol.

[22] De Ayala RJ (2009). The theory and practice of Item Response Theory.

[23] Hochberg MC (1997). Updating the American College of Rheumatology revised criteria for the classification of Systemic Lupus Erythematosus [letter]. Arthritis Rheum.

[24] World Medical Association. Declaration of Helsinki. BMJ. 1996;313(7070):1448–9.

[25] Hamashima C, Yoshida K (2001). A study of the reliability of health state valuations in the Japanese EuroQol instrument. Environ Health Prev Med.

[26] Wang S-L, Wu B, Zhu L-A, Leng L, Bucala R, Lu L-J (2014). Construct and criterion validity of the Euro Qol-5D in patients with Systemic Lupus Erythematosus. Plos One.

[27] Wright BD, Masters GN (1982). Rating scale analysis.

[28] Masters GN (1982). A Rasch model for partial credit scoring. Psychometrika.

[29] Andrich D (1988). Rasch models for measurement.

[30] Wu ML, Adams RJ, Wilson MR, Haldane SA. AcerConquest v. 3.0.1. Generalised item response modelling software. Melbourne: ACER Press; 2007.

[31] Wright BD, Linacre J, Gustafsson J, Martin-Lof P (1994). Reasonable mean-square fit values. Rasch Meas Trans.

[32] Wang W (2008). Assessment of differential item functioning. J Appl Meas.

[33] Erim Y, Kahraman Y, Vitinius F, Beckmann M, Kröncke S, Witzke O (2015). Resilience and quality of life in 161 living kidney donors before nephrectomy and in the aftermath of donation: a naturalistic single center study. BMC Nephrol.

[34] Robottom BJ, Gruber-Baldini AL, Anderson KE, Reich SG, Fishman PS, Weiner WJ (2015). What determines resilience in patients with Parkinson’s disease?. Parkinsonim Relat Disord.

[35] Schumacher A, Sauerland C, Silling G, Berdel WE, Stelljes M (2014). Resilience in patients after allogeneic stem cell transplantation. Support Care Cancer.

[36] Cal SR, Santiago MB (2013). Resilience in systemic lupus erythemathosus. Psychol Health Med.

[37] Faria DA, Revoredo LS, Vilar MJ, Chaves EM (2014). Resilience and treatment adhesion in patients with systemic lupus erythematosus. Open Rheumatol J.

